# Absent in Melanoma (AIM)2 Promotes the Outcome of Islet Transplantation by Repressing Ischemia-Induced Interferon (IFN) Signaling

**DOI:** 10.3390/cells13010016

**Published:** 2023-12-20

**Authors:** Selina Wrublewsky, Cedric Wilden, Caroline Bickelmann, Michael D. Menger, Matthias W. Laschke, Emmanuel Ampofo

**Affiliations:** Institute for Clinical & Experimental Surgery, Saarland University, 66421 Homburg, Germany; selina.wrublewsky@uks.eu (S.W.);

**Keywords:** AIM2, inflammasome, islet transplantation, interferon, revascularization

## Abstract

Clinical islet transplantation is limited by ischemia-induced islet cell death. Recently, it has been reported that the absent in melanoma (AIM)2 inflammasome is upregulated by ischemic cell death due to recognition of aberrant cytoplasmic self-dsDNA. However, it is unknown whether AIM2 determines the outcome of islet transplantation. To investigate this, isolated wild type (WT) and *AIM2*-deficient (*AIM2^−/−^*) islets were exposed to oxygen-glucose deprivation to mimic ischemia, and their viability, endocrine function, and interferon (IFN) signaling were assessed. Moreover, the revascularization and endocrine function of grafted WT and *AIM2^−/−^* islets were analyzed in the mouse dorsal skinfold chamber model and the diabetic kidney capsule model. Ischemic WT and *AIM2^−/−^* islets did not differ in their viability. However, *AIM2^−/−^* islets exhibited a higher protein level of p202, a transcriptional regulator of IFN-β and IFN-γ gene expression. Accordingly, these cytokines were upregulated in *AIM2^−/−^* islets, resulting in a suppressed gene expression and secretion of insulin. Moreover, the revascularization of *AIM2^−/−^* islet grafts was deteriorated when compared to WT controls. Furthermore, transplantation of *AIM2^−/−^* islets in diabetic mice failed to restore physiological blood glucose levels. These findings indicate that AIM2 crucially determines the engraftment and endocrine function of transplanted islets by repressing IFN signaling.

## 1. Introduction

Islet transplantation is a promising strategy to improve glycometabolic control in type 1 diabetes mellitus (T1DM) patients [1]. However, ischemia-induced islet cell death in the early post-transplant phase severely compromises the engraftment outcomes [2,3,4,5,6,7]. Therefore, numerous strategies have been developed under preclinical and clinical conditions to protect transplanted islets from ischemia-induced graft failure [8,9,10,11,12]. Particularly, approaches that prevent the deleterious events associated with apoptosis and necrosis are the focus of many studies [11,13,14]. Nonetheless, the underlying complex signaling pathways for islet cell death are still not fully understood [15].

The release of damage-associated molecular patterns (DAMP), which contain lipids, metabolites, and nucleic acids from dying islet cells, into the extracellular space is a downstream event associated with islet cell death [6,16,17]. It is well known that endogenous DAMP are recognized by inflammasomes. These multimeric protein complexes are assembled by self-oligomerizing scaffold proteins [18]. They act as a caspase-1-activation platform, which triggers immune responses and disease pathogenesis by processing pro-interleukin-1β (IL-1β) and pro-IL-18 to their mature and active forms.

The absent in melanoma (AIM)2 inflammasome belongs to the interferon (IFN)-inducible HIN200 domain-containing protein family and has been extensively studied in recent years. AIM2 is characterized by an N-terminal pyrin domain and a C-terminal HIN domain. The latter is required for binding to dsDNA in the cytosol [19,20]. Of interest, multiple AIM2 inflammasomes can bind to the same molecule of dsDNA, which potentiates its inflammatory mode of action [21]. Accordingly, AIM2 is crucial for sensing of microbial and viral DNA and, thus, alarms the innate immune system against different pathogens. Besides the well-studied function of AIM2 in the host response to pathogens [22,23,24], recent studies reported that not only hypoxia primes the formation of this inflammasome, but also self-dsDNA species released by ischemic cell death [25,26]. Moreover, ischemia and DAMP can increase the expression of IFN, which, in turn, promotes AIM2 expression [27,28].

Based on these findings, we herein speculated that AIM2 is a crucial molecular determinant for the success of islet transplantation. To clarify this, we exposed in vitro wild type (WT) and *AIM2*-deficient (*AIM2^−/−^*) islets to oxygen-glucose deprivation to mimic ischemia and analyzed their viability, endocrine function, and interferon (IFN) signaling. In addition, we assessed their in vivo revascularization and endocrine function in the mouse dorsal skinfold chamber model and the diabetic kidney capsule model.

## 2. Material and Methods

### 2.1. Material

QIAzol was purchased from Qiagen (Hilden, Germany). Bovine serum albumin (BSA) was purchased from Santa Cruz Biotechnology (Heidelberg, Germany). Roswell Park Memorial Institute (RPMI)-1640 medium, Dulbecco’s Modified Eagle’s Medium (DMEM), IFN-β, and IFN-γ were purchased from Thermo Fisher Scientific (Karlsruhe, Germany). The qScriber cDNA Synthesis Kit and ORA SEE qPCR Green ROX L Mix were purchased from HighQu (Kraichtal, Germany). The Collagenase NB 8 Broad Range was purchased from Nordmark Biochemicals (Uetersen, Germany). HepatoQuick^®^, Cell Proliferation Reagent water-soluble tetrazolium (WST)-1, Cytotoxicity Detection Kit^PLUS^, and Annexin-V-Fluos Staining Kit were purchased from Roche (Basel, Switzerland). Matrigel was purchased from Corning (Wiesbaden, Germany). Polyvinylidene difluoride (PVDF) membrane was purchased from Bio-Rad (Feldkirchen, Germany). Accutase was purchased from BioLegend (Koblenz, Germany). Propidium iodide was purchased from BD Biosciences (Heidelberg, Germany). Hematoxylin was purchased from Morphisto (Offenbach am Main, Germany). Tween20, Hoechst 33342, Fluorescein-isothiocyanate (FITC)-labeled dextran 150,000, neutral red solution, rhodamine 6G, penicillin, and streptozotocin (STZ) were purchased from Sigma-Aldrich (Taufkirchen, Germany).

### 2.2. Antibodies

The anti-insulin (ab181547), anti-somatostatin (ab30788), anti-glucagon (ab92587), anti-MPO (ab9535), anti-CD3 (ab16669), and anti-CD68 (ab125212) antibodies were purchased from Abcam (Cambridge, UK). The anti-CD31 antibody (DIA310) was purchased from Dianova (Hamburg, Germany). The anti-p202 (PA5-23483) was purchased from Invitrogen (Carlsbad, CA, USA). The anti-rabbit IgG Alexa Fluor 555 (A-21429) and anti-rat IgG Alexa Fluor 488 (A-21434) antibodies were purchased from Thermo Fisher Scientific (Karlsruhe, Germany). The secondary antibodies (anti-rabbit (NIF 824)) and peroxidase-labeled anti-mouse (NIF 825)) were purchased from GE Healthcare (Freiburg, Germany).

### 2.3. Cell Culture

Human umbilical vein endothelial cells (HUVEC) were cultivated in Endothelial Cell Basal Medium (100 U/mL penicillin and 0.1 mg/mL streptomycin) at 37 °C under a humidified 95% to 5% (*v*/*v*) mixture of air and CO_2_. HUVEC were passaged at a split ratio of 1:3 after reaching confluence.

### 2.4. WST-1 Assay

HUVEC were seeded in a 96-well plate and treated with vehicle (0.1% BSA), IFN-β (10, 50, 100, 150, and 200 ng/mL dissolved in 0.1% BSA), or IFN-γ (10, 50, 100, 150, and 200 ng/mL dissolved in 0.1% BSA) for 24 h. Thereafter, a WST-1 assay was used to measure the metabolic activity of treated HUVEC, and it was carried out according to the manufacturer’s instructions.

### 2.5. Lactate Dehydrogenase (LDH) Assay

HUVEC were treated with vehicle (0.1% BSA), IFN-β (10, 50, 100, 150, and 200 ng/mL dissolved in 0.1% BSA), or IFN-γ (10, 50, 100, 150, and 200 ng/mL dissolved in 0.1% BSA) for 24 h. Thereafter, a LDH assay was used to analyze the cytotoxic effects of IFN-β and IFN-γ on HUVEC according to the manufacturer’s instructions.

### 2.6. Tube Formation Assay

HUVEC were seeded in a 96-well plate (1.5 × 10^4^ cells per well), which contained 50 µL Matrigel per well. The cells were exposed to IFN-β (100 ng/mL dissolved in 0.1% BSA), IFN-γ (100 ng/mL dissolved in 0.1% BSA), or vehicle (0.1% BSA). Phase-contrast light images were taken after 5 h and 7 h. Tube formation was quantified by means of measuring the number of tube meshes using FIJI software (U.S. NIH).

### 2.7. Animals

Animals were maintained on a standard 12/12 h day/night cycle. Water and standard pellet chow (Altromin, Lage, Germany) were provided ad libitum. Transgenic *AIM2*^−/−^ (B6.129P2-Aim2^Gt(CSG445)Byg^/J) mice were purchased from the Jackson Laboratory. C57BL/6J WT and *AIM2*^−/−^ mice with a body weight of 25–30 g were used as donors for islet isolation. We used C57BL/6J WT mice (body weight of 23–25 g) for the dorsal skinfold chamber model. To induce a diabetic phenotype, we used male C57BL/6J WT (body weight of 24–28 g).

The in vivo experiments were carried out according to the German legislation on protection of animals and the National Institutes of Health (NIH) Guide for the Care and Use of Laboratory Animals (Institute of Laboratory Animal Resources, National Research Council, Washington, DC, USA). The experiments were approved by the local governmental animal protection committee (permission number: 45/2018).

### 2.8. Isolation of Pancreatic Islets

Mice were sacrificed by cervical dislocation, and pancreatic islets were isolated as described previously in detail [29]. Isolated islets were cultivated in RPMI-1640 medium (supplemented with 10% (*v*/*v*) fetal calf serum, 100 U/mL penicillin, and 0.1 mg/mL streptomycin) for 24 h at 37 °C and 5% CO_2_ for further experiments.

### 2.9. Oxygen-Glucose Deprivation

Isolated WT, *AIM2*^−/−^, and WT islets exposed to IFN-β (50 ng/mL dissolved in 0.1% BSA), IFN-γ (50 ng/mL dissolved in 0.1% BSA), or vehicle (0.1% BSA) were cultivated in DMEM (1 g/L glucose) under hypoxic conditions (95% N_2_, 5% CO_2_, and 5% O_2_) for 48 h to mimic ischemia in vitro.

### 2.10. Propidium Iodide/Annexin V Staining

Ischemic WT and *AIM2^−/−^* islets were dispersed into single cells by accutase. Subsequently, the cells were washed in phosphate-buffered saline (PBS) and stained with propidium iodide and annexin V (100 μg/mL) according to the manufacturer’s protocol (Roche). Islet cells were determined by means of flow cytometry (FACSLyrics (BD Biosciences)) and the fraction of vital, apoptotic, necroptotic as well as necrotic cells were given in % of all measured cells.

### 2.11. Western Blot Analysis

Cell extracts of ischemic WT and *AIM2*^−/−^ islets were generated, as described previously in detail [30]. Protein extracts were separated through a 12.5% SDS polyacrylamide gel and subsequently transferred onto a PVDF membrane. This was exposed to anti-p202 and anti-β-actin antibodies (diluted (1:500) in PBS (0.1% Tween20) containing 1% dry milk). Thereafter, the membrane was incubated with a secondary antibody, and the proteins were visualized by enhanced chemoluminescence (ECL) Western blotting substrate (GE Healthcare) in a Chemocam device (Intas; Göttingen, Germany).

### 2.12. Quantitative Real-Time Polymerase Chain Reaction (qRT-PCR)

Total RNA from ischemic WT and *AIM2*^−/−^ islets was isolated using QIAzol lysis reagent (Qiagen). The corresponding cDNA was synthesized by QuantiNova Reverse Transcription Kit (Qiagen) as described in the manufacturer’s instructions. We used ORA qPCR Green ROX L Mix (highQu) for qRT-PCR, and the data analysis was carried out by the MiniOpticon Real-Time PCR System (Bio-Rad). Murine β-actin was used as the control. Forward and reverse primers (solved in RNase/DNase-free H_2_O) were used in a concentration of 700 nM. Primer sequences for qPCR were coded as follows: Mouse IFN-β forward 5′-GCCTTTGCCATCCAAGAGATGC-3′; reverse 5′-ACACTGTCTGCTGGTGGAGTTC-3′; Mouse IFN-γ forward 5′-CAGCAACAGCAAGGCGAAAAAGG-3′; reverse 5′-TTTCCGCTTCCTGAGGCTGGAT-3′; Mouse Ins-1 forward 5′-AACAACTGGAGCTGGGAGGAAG-3′, reverse 5′-GGTGCAGCACTGATCCACAATG-3′; Mouse Ins-2 forward 5′-GCAGCACCTTTGTGGTTCC-3′, reverse 5′-CTTGTGGGTCCTCCACTTC-3′; Mouse β-actin forward 5′-AGCCTTCCTTCTTGGGTATGG-3′, reverse 5′-CGGATGTCAACGTCACACTTC-3′.

### 2.13. Enzyme-Linked Immunosorbent Assay (ELISA)

Released insulin was measured by an insulin ELISA. For this purpose, 10 isolated islets were washed with Krebs Ringer buffer (KRB; 115 mM NaCl, 4.7 mM KCl, 1.28 mM CaCl_2_, 1.2 mM MgSO_4_, 0.1% BSA) and incubated for 1 h at 37 °C and 5% CO_2_. The supernatants were discarded, and the islets were incubated for 30 min in KRB containing 16.5 mM glucose. The supernatants were collected, and the amount of secreted insulin was determined by using an insulin ELISA kit (Invitrogen) according to the manufacturer’s protocol.

### 2.14. Preparation of the Dorsal Skinfold Chamber and Islet Transplantation

The dorsal skinfold chamber was implanted, as described previously in detail [31]. Briefly, two symmetrical titanium frames were prepared on the extended dorsal skinfold of anesthetized mice to double the skin in two layers. One layer was removed in a circular area of 15 mm in diameter. The area was covered by a cover slip as well as a snap ring. After this procedure, all animals were allowed to recover for 48 h. Then, the cover glass was removed, and the striated muscle tissue was washed with saline. Eight islets were transplanted onto the exposed tissue, and the chamber was sealed with a cover slip.

### 2.15. Intravital Fluorescence Microscopy

Anesthetized (intraperitoneal injection of ketamine (100 mg/kg body weight) and xylazine (12 mg/kg body weight)) dorsal skinfold chamber-equipped mice received a retrobulbary intravenous injection of 0.05 mL FITC-labeled dextran (5%) and 0.05 mL rhodamine 6G (2%) [8]. The microscopic images were recorded for off-line evaluation by the computer-assisted image analysis system CapImage (Zeintl, Heidelberg, Germany). The revascularized area (mm^2^), the functional microvessel density (cm/cm^2^) as well as the rhodamine 6G-positive area (calculated by the ratio of the rhodamine 6G-positive area and the revascularized area on that day in %) of islets were assessed, as previously described [8]. Furthermore, we measured the diameter (µm), centerline red blood cell (RBC) velocity (µm/s), and volumetric blood flow (pL/s) of 4–8 individual microvessels within the transplants [8]. The take rate (%), i.e., the number of engrafted islets on day 14 in relation to the number of transplanted islets on day 0, was determined.

### 2.16. Immunohistochemistry

The pancreas of WT and *AIM2*^−/−^ mice was excised and fixed for 24 h by means of 4% paraformaldehyde (PFA). In addition, isolated islets from WT and *AIM2*^−/−^ donor mice were incubated for 45 min at 37 °C in 100 µL HepatoQuick^®^, 50 µL human citrate plasma, and 10 µL 10% CaCl_2_ solution. The resulting clot was also fixed for 24 h in 4% PFA. The PFA-fixed specimens were embedded in paraffin and 3-μm-thick sections were cut.

Then, the sections were stained with antibodies against insulin (1:300), glucagon (1:300), somatostatin (1:300), CD31 (1:300), MPO (1:300), CD68 (1:300), and CD3 (1:300) and visualized by secondary antibodies. We used Hoechst 33342 and hematoxylin to stain cell nuclei. The sections were examined by means of a BX60F microscope (Olympus). The quantification was carried out by FIJI software (NIH) and is given in % of all islet cells.

### 2.17. Diabetes Induction and Islet Transplantation under the Kidney Capsule

Diabetes was induced by an i.p. injection of 180 mg/kg STZ for 8 days. Body weights and non-fasting blood glucose levels of STZ-induced diabetic mice were analyzed twice a week. Blood samples were taken from the tail vein, and the blood glucose level was assessed by means of a blood glucose monitoring system (GL50; Breuer). For islet transplantation, we used only mice with a non-fasting blood glucose level ≥ 350 mg/dL. We injected 400 isolated islets under the left kidney capsule of diabetic recipients via a 10 µL Hamilton syringe. Blood glucose levels below 200 mg/dL were defined as normoglycemia.

### 2.18. Intraperitoneal Glucose Tolerance Test (IPGTT) and Insulin Content

We performed an IPGTT on day 28 after islet transplantation. After 16 h of fasting, the mice were i.p. injected with a 10% glucose solution. Glucose levels in blood samples from the tail vein were determined 0, 15, 30, 45, 60, 120, and 180 min after glucose injection and analyzed by a portable blood glucose monitoring system (GL50; Breuer).

To analyze the total insulin content of the transplants, we excised the islet transplants underneath the kidney capsule. Then, we lysed them and determined the insulin content via an insulin ELISA kit.

### 2.19. Statistical Analysis

The in vitro experiments were repeated at least 3 times. We used at least 6 mice per group for the in vivo studies. After testing the data for normal distribution and equal variance, we assessed the differences between 2 groups by the unpaired Student’s *t*-test. To test differences between multiple groups, we used one-way ANOVA followed by the Tukey post-hoc test. All statistical analyses were performed by means of Prism software 8 (GraphPad). All values are expressed as mean ± SEM. Statistical significance was accepted for *p* < 0.05.

## 3. Results

### 3.1. AIM2 Deficiency Does Not Affect the Cellular Composition of Isolated Islets

**Figure 1:**
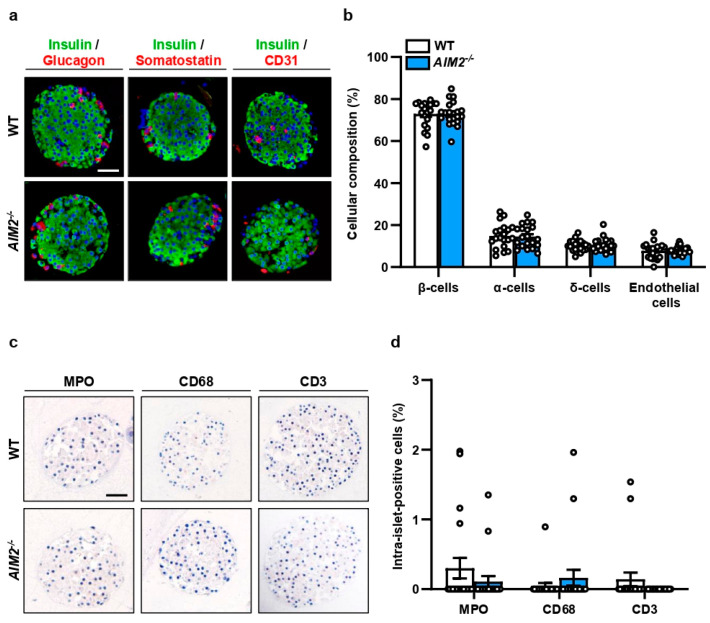
*AIM2* deficiency does not affect the cellular composition of isolated islets under normoxia. (**a**) Double stainings of insulin/glucagon, insulin/somatostatin, and insulin/CD31 in isolated WT and *AIM2^−/−^* islets. Hoechst 33342 (blue) was used to stain cell nuclei. Scale bar: 50 µm. (**b**) Insulin- (β-cells), glucagon- (α-cells), somatostatin- (δ-cells), and CD31- (endothelial) positive cells (in % of all islet cells) in isolated WT and *AIM2^−/−^* islets were quantitatively analyzed (n = 20 each). Mean ± SEM. (**c**) Stainings of MPO-, CD68- and CD3-positive cells in isolated WT and *AIM2^−/−^* islets. Scale bar: 50 µm. (**d**) MPO-, CD68- and CD3-positive cells (in % of all islet cells) in isolated WT and *AIM2^−/−^* islets were quantitatively analyzed (n = 20 each). Mean ± SEM.

We first studied the cellular composition of islets within the pancreas of WT and *AIM2^−/−^* mice as well as of isolated WT and *AIM2^−/−^* islets by immunohistochemical stainings of insulin-, glucagon-, somatostatin-, and CD31-positive cells (Supplementary Figure S1a; Figure 1a). We found no differences between the groups (Supplementary Figure S1b; Figure 1b). We additionally investigated the fractions of islet resident MPO-positive neutrophilic granulocytes, CD68-positive macrophages, and CD3-positive lymphocytes (Supplementary Figure S1c; Figure 1c). As expected, we detected similar amounts of the different immune cell subtypes in the two groups (Supplementary Figure S1d; Figure 1d).

### 3.2. AIM2 Deficiency in Ischemic Islets Reduces Insulin Secretion

**Figure 2:**
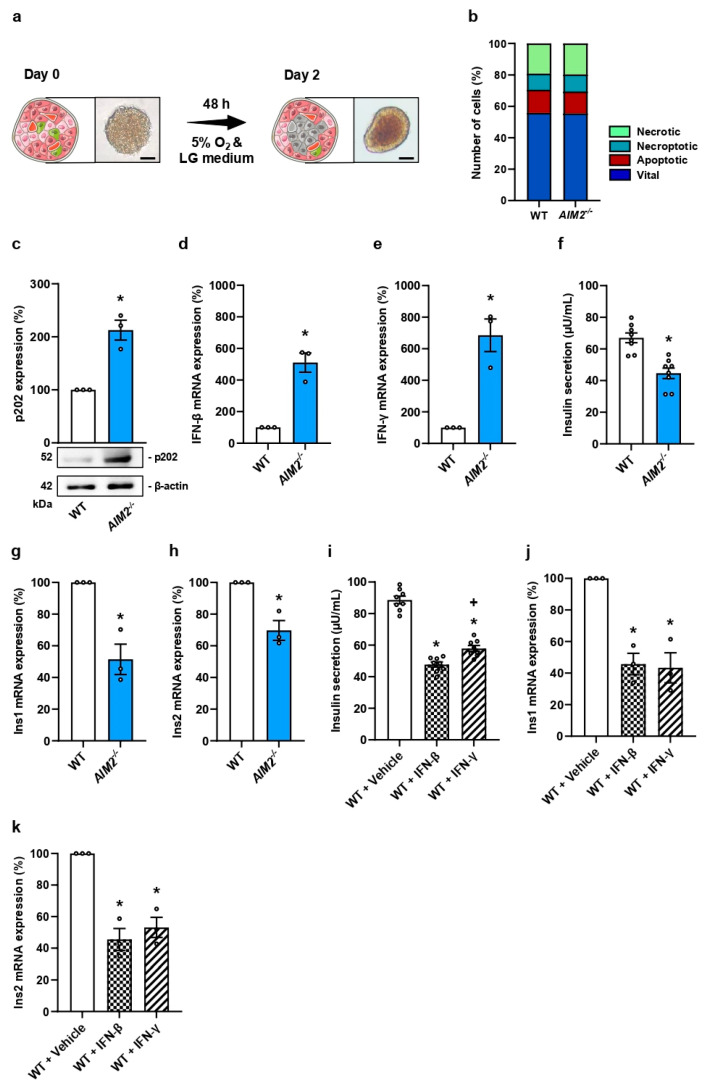
*AIM2* deficiency reduces insulin expression in an IFN-dependent manner. (**a**) Schematic illustration of the experimental in vitro setting. Freshly isolated islets (left: bright field microscopic image; scale bar: 75 µm) were exposed to hypoxia (5% O_2_) and low glucose (LG) medium (1 g/L glucose) for 48 h to induce central cell death within the islets (right: bright field microscopic image; scale bar: 75 µm). These ischemic islets were used for further experiments. (**b**) Propidium iodide/annexin V-stained cells (in % of total cell number) were quantitatively analyzed in ischemic WT and *AIM2^−/−^* islets subdivided in necrotic, necroptotic, apoptotic, and vital cells (n = 3 each). (**c**) Representative Western blot of p202 expression from extracts of ischemic WT and *AIM2^−/−^* islets (lower panels). Quantitative analysis of p202 expression (upper panel). β-actin was used as loading control. Data are expressed in % of WT (n = 3 each). Mean ± SEM. * *p* < 0.05 vs. WT. (**d**,**e**) IFN-β (**d**) and IFN-γ (**e**) mRNA expression in ischemic WT and *AIM2^−/−^* islets were quantitatively analyzed. Data are expressed in % of WT (n = 3 each). Mean ± SEM. * *p* < 0.05 vs. WT. (**f**) Quantitative analysis of insulin secretion (µU/mL) from ischemic WT and *AIM2^−/−^* islets (n = 8 each). Mean ± SEM. * *p* < 0.05 vs. WT. (**g**,**h**) Ins1 (**g**) and Ins2 (**h**) mRNA expression in ischemic WT and *AIM2^−/−^* islets were quantitatively analyzed. Data are expressed in % of WT (n = 3 each). Mean ± SEM. * *p* < 0.05 vs. WT. (**i**) Quantitative analysis of insulin secretion (µU/mL) from ischemic WT islets exposed to vehicle, IFN-β, or IFN-γ (n = 8 each). Mean ± SEM. * *p* < 0.05 vs. WT + Vehicle; ^+^ *p* < 0.05 vs. WT + IFN-β. (**j**,**k**) Quantitative analysis of Ins1 (**j**) and Ins2 (**k**) mRNA expression in ischemic WT islets exposed to vehicle, IFN-β, or IFN-γ. Data are expressed in % of WT + Vehicle (n = 3 each). Mean ± SEM. * *p* < 0.05 vs. WT + Vehicle.

To mimic in vitro ischemia-induced islet cell death, we cultivated WT and *AIM2^−/−^* islets in a low glucose medium under hypoxic conditions for 48 h and subsequently measured the number of vital, apoptotic, necroptotic, and necrotic cells (Figure 2a,b). Bright field microscopy clearly showed dead cells in the center of the cultivated islets exposed to oxygen-glucose deprivation (Figure 2a). The analysis of the underlying cell death mechanisms revealed caspase-dependent apoptosis and caspase-independent necrosis (Figure 2b). Of note, the loss of AIM2 did not protect islet cells from ischemia-induced cell death (Figure 2b).

*AIM2* deficiency is inversely correlated with the expression of IFN-β, IFN-γ, and IFN-inducible genes, such as p202 [32,33]. Moreover, it is well known that IFN impairs insulin secretion [34,35,36]. In line with these findings, we could show that AIM2 deficiency increases p202, IFN-β, and IFN-γ expression in ischemic islets (Figure 2c–e). Furthermore, we detected a reduced glucose-stimulated insulin secretion (GSIS) in ischemic *AIM2^−/−^* islets when compared to WT controls (Figure 2f), which was caused by a decreased insulin gene expression (Figure 2g,h). To prove that this effect is largely mediated by IFN-β and IFN-γ, we determined GSIS and insulin expression of ischemic WT islets exposed to both cytokines (Figure 2i–k). As expected, IFN-β and IFN-γ significantly decreased the secretion and expression of insulin (Figure 2i–k).

### 3.3. AIM2 Deficiency Impairs the Revascularization of Transplanted Islets

**Figure 3:**
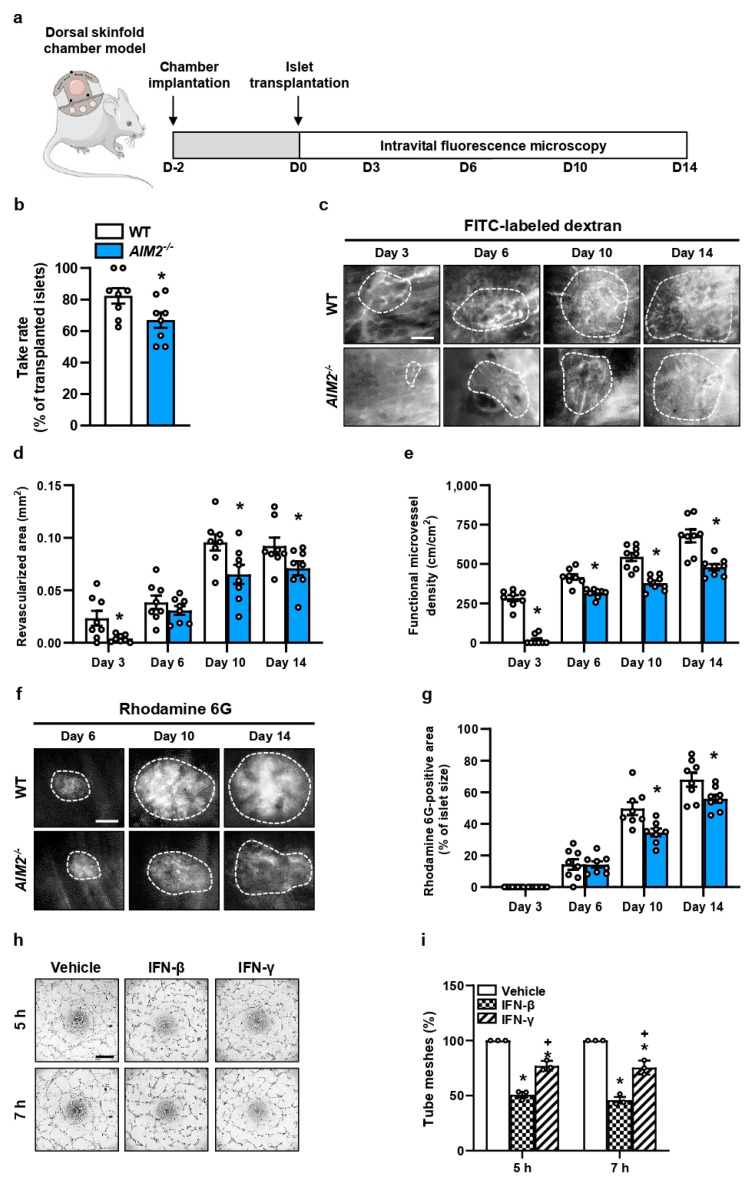
*AIM2* deficiency impairs the graft revascularization. (**a**) Experimental in vivo setting. On day -2, we implanted the dorsal skinfold chambers. WT and *AIM2^−/−^* islets were transplanted on day 0. Intravital fluorescence microscopy was performed on day 0, 3, 6, 10, and 14 after islet transplantation. (**b**) Take rate of WT and *AIM2^−/−^* islets (% of transplanted islets; n = 8 each) on day 14. Mean ± SEM. * *p* < 0.05 vs. WT. (**c**) Intravital fluorescent microscopic images of transplanted WT and *AIM2^−/−^* islets within the dorsal skinfold chamber. Blood-perfused microvessels are stained with FITC-labeled dextran. The border of the grafts is marked by broken lines. Scale bar: 100 μm. (**d**) The revascularized area (mm^2^) of WT and *AIM2^−/−^* islets was quantitatively analyzed in (n = 8 each). Mean ± SEM. * *p* < 0.05 vs. WT. (**e**) The functional microvessel density (cm/cm^2^) of WT and *AIM2^−/−^* islets was quantitatively analyzed in (n = 8 each). Mean ± SEM. * *p* < 0.05 vs. WT. (**f**) Intravital fluorescent microscopic images of transplanted WT and *AIM2^−/−^* islets within the dorsal skinfold chamber. We used Rhodamine 6G to stain the perfusion of the endocrine tissue (bright signals). Broken lines mark the border of the transplants. Scale bar: 100 µm. (**g**) Quantitative analysis of the rhodamine 6G-positive area (% of islet size) within transplanted WT and *AIM2^−/−^* islets (n = 8 each). Mean ± SEM. * *p* < 0.05 vs. WT. (**h**) Tube formation assays were performed with HUVEC exposed to vehicle, IFN-β, or IFN-γ. The formation of vessel-like structures was analyzed 5 h and 7 h after seeding. Scale bar: 750 µm. (**i**) Quantitative analysis of the number of tube meshes (% of vehicle) after 5 h and 7 h (n = 3 each). Mean ± SEM. * *p* < 0.05 vs. Vehicle; ^+^ *p* < 0.05 vs. IFN-β.

We studied the development of novel blood vessels in transplanted WT and *AIM2^−/−^* islets by intravital fluorescence microscopy (Figure 3a). Our results showed that the loss of AIM2 significantly decreases the take rate of the grafts, i.e., the number of engrafted islets on day 14 in relation to the overall number of transplanted islets per group (Figure 3b). In line with these results, we observed a lower revascularized area and functional microvessel density throughout the entire observation period in grafted *AIM2^−/−^* islets when compared to WT controls (Figure 3c–e). Furthermore, we detected a smaller rhodamine 6G-positive area within *AIM2^−/−^* islets, indicating a reduced endocrine tissue perfusion (Figure 3f,g). The loss of AIM2 does not affect microhemodynamic parameters such as the diameter, centerline RBC velocity, and volumetric blood flow of intra-islet blood vessels (Supplementary Figure S2a–c).

IFN signaling has been shown to reduce the angiogenic activity of endothelial cells [37,38]. To elucidate that the impaired engraftment of *AIM2^−/−^* islets is mediated by IFN signaling, we performed a tube formation assay (Figure 3h,i). This assay clearly showed that the exposure of HUVEC to IFN-β or IFN-γ deteriorates angiogenic tube formation when compared to vehicle-treated endothelial cells (Figure 3i). Additional WST-1 and LDH assays revealed that this effect was not caused by a reduced cell viability (Supplementary Figure S3a,b). These findings support the assumption that the elevated IFN-β and IFN-γ levels in ischemic *AIM2^−/−^* islets inhibited their revascularization after transplantation.

### 3.4. AIM2 Deficiency in Transplanted Islets Impairs Restoration of Normoglycemia in Diabetic Mice

**Figure 4:**
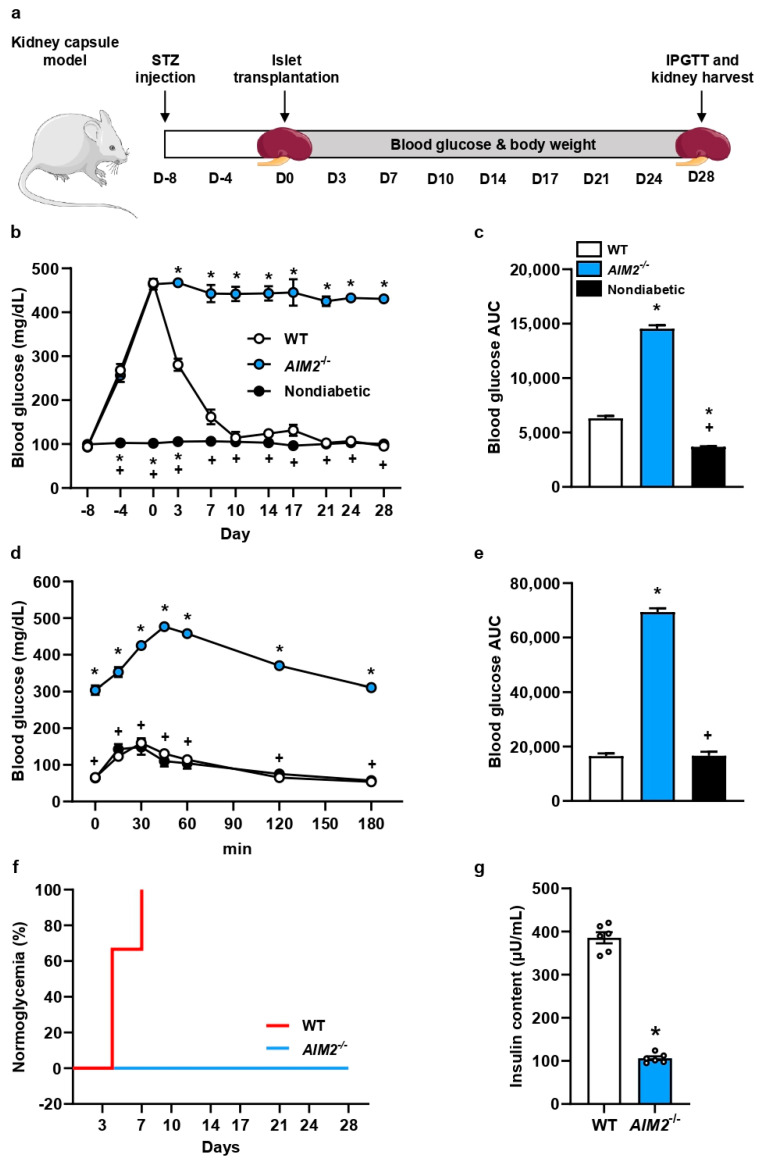
*AIM2* deficiency in transplanted islets impairs restoration of normoglycemia in diabetic mice. (**a**) Experimental in vivo setting. Eight days prior to islet transplantation, we induced a diabetic phenotype by STZ injection. On day 0, 400 islets were transplanted under the kidney capsule of diabetic recipient mice. Subsequently, we measured body weights and blood glucose levels twice a week. On day 28, we performed an IPGTT and excised the kidneys to determine the insulin content of the transplants. (**b**) Blood glucose levels (mg/mL) of diabetic recipient mice transplanted with WT and *AIM2^−/−^* islets from day -8 to day 28 (n = 6 each). Nondiabetic animals were used as negative control (n = 6 each). Mean ± SEM. * *p* < 0.05 vs. WT; ^+^ *p* < 0.05 vs. *AIM2^−/−^*. (**c**) AUC of the blood glucose levels from (**b**) (n = 6 each). Mean ± SEM. * *p* < 0.05 vs. WT; ^+^ *p* < 0.05 vs. *AIM2^−/−^*. (**d**) Quantitative analysis of blood glucose levels (mg/dL) on day 28 according to the IPGTT of diabetic recipient mice transplanted with WT and *AIM2^−/−^* islets (n = 6 each). Nondiabetic animals were used as negative control (n = 6 each). Mean ± SEM. * *p* < 0.05 vs. WT; ^+^ *p* < 0.05 vs. *AIM2^−/−^*. (**e**) AUC of IPGTT from (**d**) (n = 6 each). Mean ± SEM. * *p* < 0.05 vs. WT; ^+^ *p* < 0.05 vs. *AIM2^−/−^*. (**f**) The fraction of animals achieving physiological blood glucose levels (n = 6 each). (**g**) Insulin content (µU/mL) of the removed grafts from diabetic mice transplanted with WT and *AIM2^−/−^* islets (n = 6 each). Mean ± SEM. * *p* < 0.05 vs. WT.

Next, we examined whether the loss of AIM2 in transplanted islets also leads to an impaired restoration of normoglycemia in diabetic recipients. For this, 400 islets were transplanted under the kidney capsule of STZ-induced diabetic mice, and blood glucose levels as well as body weights were analyzed (Figure 4a). Nondiabetic animals served as the negative control. We measured markedly lower blood glucose levels in mice transplanted with WT islets 3 days after transplantation when compared to mice receiving *AIM2^−/−^* islets (Figure 4b). In fact, the transplantation of *AIM2^−/−^* islets did not restore normoglycemia during the entire 28-day observation period (Figure 4b). As expected, the area under the curve (AUC) of the *AIM2^−/−^* group was significantly higher compared to the WT and nondiabetic group (Figure 4c). The body weights of the animals receiving *AIM2^−/−^* islets were lower when compared to both control groups (Supplementary Figure S4a,b). An IPGTT further revealed that blood glucose levels of mice transplanted with *AIM2^−/−^* islets were significantly higher when compared to control animals (Figure 4d,e). Of interest, transplanted WT islets reversed diabetes in 100% of recipient mice on day 7, whereas none of the mice receiving *AIM2^−/−^* islets were normoglycemic on day 28 (Figure 4f). Furthermore, we also determined lower plasma insulin levels in the *AIM2^−/−^* group 15 min after glucose injection (Figure 4g).

## 4. Discussion

Several studies have already demonstrated that the inhibition of the nucleotide-binding oligomerization domain (NOD)-like receptor protein (NLRP)3 inflammasome, which is the most studied inflammasome sensor due to its involvement in pathogen-driven and sterile inflammation, improves the endocrine function of islets [39,40,41,42]. In line with this observation, we have recently shown that downregulation of NLRP3 activity in isolated islets prior to transplantation accelerates their engraftment due to an increased insulin gene expression [42]. However, whether the inhibition of other inflammasomes also promotes islet transplantation is still not known. To close this knowledge gap, we analyzed the effect of AIM2 on the endocrine and angiogenic function of transplanted islets.

Pancreatic islets are surrounded by a dense capillary network that guaranties the release of endocrine hormones and the supply of nutrients and oxygen [3,8,43]. However, the enzymatic isolation of islets destroys this capillary network, and the islets have to be revascularized immediately after transplantation [8]. Until the completion of this revascularization process, islets are exposed to ischemia, which results in massive islet cell death [15,44,45]. Genaro et al. [6] reported that ischemic islets release DAMP, including high mobility group box-1 (HMGB1), uric acid as well as dsDNA as a downstream event of necrotic and apoptotic islet cell death. Of note, self-dsDNA can trigger the immune response by activating AIM2 [46]. Therefore, we assumed that the AIM2 inflammasome is particularly involved in islet graft failure during the early post-transplant phase. To assess ischemia-induced β-cell death in vitro, we exposed isolated islets to defined oxygen and nutrient conditions (5% O_2_, 1 g/L glucose, no serum) that mimic the microenvironment shortly after transplantation, as shown by massive cell death in the center of the islets.

It has been demonstrated that AIM2 triggers apoptosis by activation of caspase-8 and -1 [19]. In addition, we detected an elevated protein level of the IFN-inducible protein p202 in ischemic *AIM2^−/−^* islets. This protein is a potential inhibitor of AIM2 and has been reported to inhibit apoptosis [32,47]. Accordingly, loss of the AIM2 inflammasome may protect islets from ischemia-induced cell death. However, contrary to this assumption, we detected similar numbers of apoptotic, necrotic, and necroptotic cells in ischemic WT and *AIM2^−/−^* islets. This may be explained by the increased expression of IFN-β and IFN-γ in ischemic *AIM2^−/−^* islets, because IFN signaling has been shown to promote cell death in multiple ways [48]. Hence, these cytokines may have compensated the protective effects of *AIM2* deficiency on islet cell viability.

We further analyzed the effect of AIM2 on the endocrine function of pancreatic islets. Our results demonstrated a decreased insulin expression and secretion in ischemic *AIM2^−/−^* islets. It is well known that IFN signaling impairs the endocrine function of pancreatic islets [34,35,36]. Accordingly, the herein observed reduced insulin secretion after loss of AIM2 may be caused by the upregulated expression of IFN-β and IFN-γ. In fact, the exposure of ischemic WT islets to both cytokines markedly attenuated insulin expression and secretion. It is well known that insulin as well as pro-inflammatory cytokines trigger angiogenic processes. In this context, it has already been shown that the binding of insulin to insulin-like insulin receptor (IR) kinase and the insulin growth factor-1 receptor (IGF-1R) of endothelial cells promotes revascularization of the grafts [49,50]. Furthermore, cytokines such as interleukin-6 stimulate angiogenesis [51] and, thus, improve the outcome islet transplantation [52]. We herein found a lower take rate of transplanted islets in the *AIM2^−/−^* group, which was due to a reduced graft revascularization. In line with this result, Cui et al. [53] could show in a model of laser-induced choroidal neovascularization that the loss of AIM2 significantly inhibits vascular leakage, blood vessel formation, and lesion thickness [53]. These findings could be explained by the higher levels of IFN-β/γ, which have been demonstrated to repress angiogenesis [37,38,54]. Finally, we transplanted a minimal mass of *AIM2*-deficient islets under the kidney capsule of diabetic recipient mice. Mice receiving *AIM2^−/−^* islets did not exhibit normoglycemia over the entire observation period when compared to mice receiving WT islets. This could be explained by the reduced revascularization and, thus, lower number of grafted islets as well as by the decreased insulin expression and secretion in the *AIM2^−/−^* group. These findings clearly demonstrate the essential role of AIM2 for the endocrine function and revascularization capacity of transplanted islets.

AIM2 is highly expressed in myeloid-derived cells [18,20,55]. For instance, treatment of the HL-60 cell line with IFN-γ [56] or exposure of macrophages to IFN-β markedly upregulates AIM2 expression [57]. Leite et al. [58] detected lower serum levels of insulin in *AIM2*-deficient diabetic mice when compared to diabetic control animals. Moreover, they could show that mainly CD3-positive lymphoid and CD11b-positive myeloid cells express AIM2 [58]. Interestingly, isolated islets contain a small fraction of resident macrophages [59], which are the main source of inflammatory cytokines, such as IFN [60]. Therefore, we speculate that the herein observed effects of AIM2 deficiency on the function of islet grafts are triggered by the elevated IFN-β and IFN-γ expression of islet-resident macrophages.

It is well known that the inhibition of inflammasomes reduces the progression of various sterile inflammatory diseases such as kidney diseases, cardiovascular diseases, and neuronal diseases [61,62]. In line with this view, it has been shown that the specific inhibition of the NLRP3 inflammasome also decreases the progression of T2DM by increasing insulin secretion. More importantly, we could demonstrate that the treatment of islets with CY-09, a specific NLRP3 inhibitor, prior to their transplantation improves the outcome of islet transplantation. Soriano-Teruel et al. [63] recently introduced MM01 as a novel broad-spectrum inflammasome inhibitor for the treatment of multifactorial diseases. Accordingly, this inhibitor may improve the success of islet transplantation. However, the results of the present study clearly show that AIM2 inhibition deteriorates the success of islet transplantation. Hence, the use of pan-inflammasome inhibitors to improve islet transplantation must be carefully evaluated in the future.

In summary, we found that the loss of AIM2 in isolated islets markedly impairs the outcome of islet transplantation. Analyses of the underlying mechanisms revealed that this is most probably due to an IFN-induced repression of the endocrine function and angiogenic activity of pancreatic islets.

## Data Availability

The data presented in this study are available on request from the corresponding author.

## Supplementary Materials

The following supporting information can be downloaded at: https://www.mdpi.com/article/10.3390/cells13010016/s1, Figure S1: Cellular composition of islets within the pancreas; Figure S2: Microhemodynamic parameters of transplanted islets; Figure S3: Effect of IFN-β and IFN-γ on the proliferation of HUVEC; Figure S4: Body weights of islet transplanted mice.
