# Supplementary material for: Absent in Melanoma (AIM)2 Promotes the Outcome of Islet Transplantation by Repressing Ischemia-Induced Interferon (IFN) Signaling
Source: Cells. 2023 Dec 20;13(1):16. doi: 10.3390/cells13010016 (PMC10778091; doi:10.3390/cells13010016)
Supplement: Supplementary file 1 [file cells-13-00016-s001.zip › cells-2737462-supplementary.pdf]

Figure S1

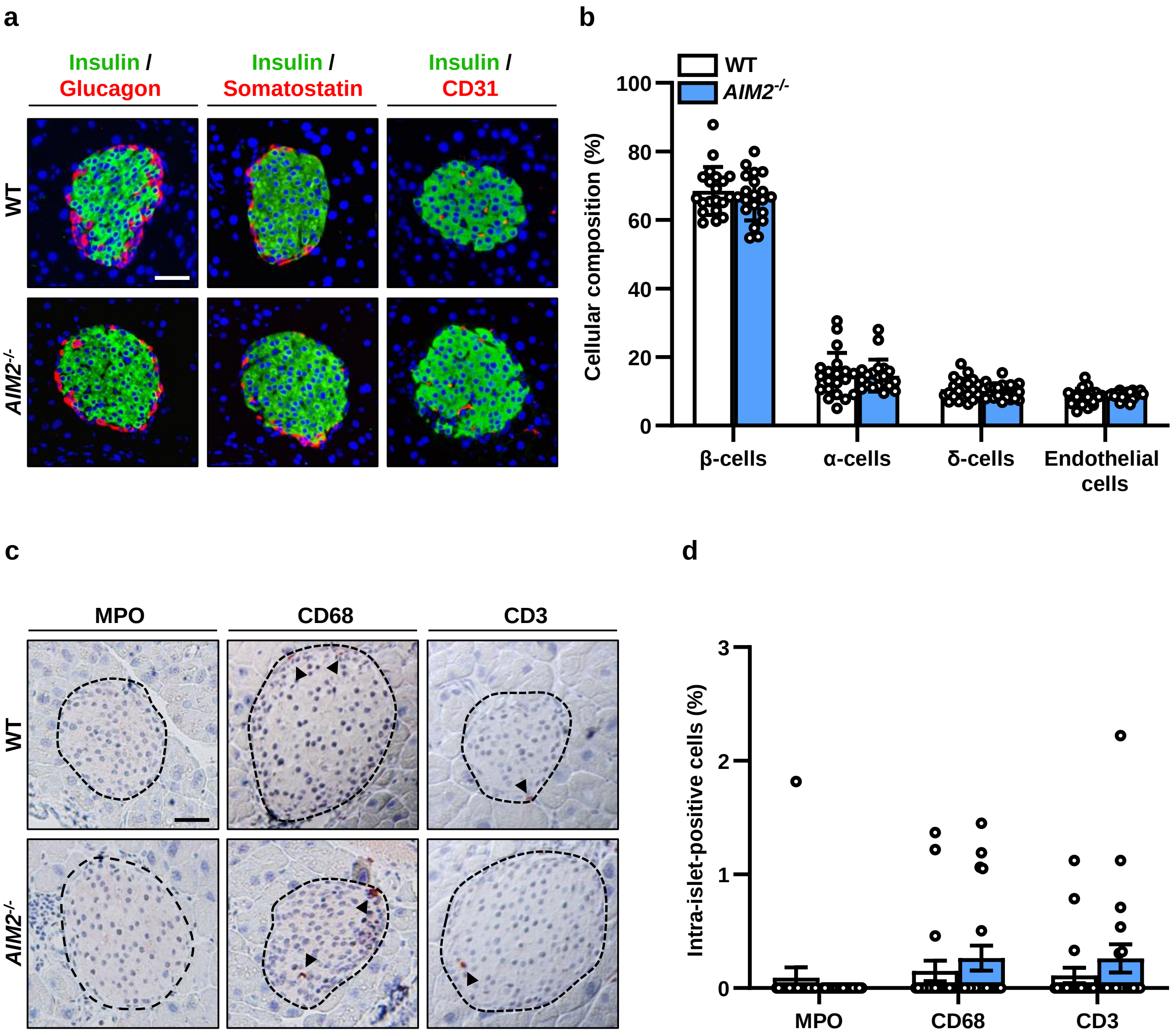

Figure S1. Cellular composition of islets within the pancreas. (**a**) Representative immunofluorescence stainings of insulin/glucagon, insulin/somatostatin and insulin/CD31 in WT and *AIM2*$^{-/-}$ islets within the pancreas. Cell nuclei were stained with Hoechst 33342 (blue). Scale bar: 50 µm. (**b**) Quantitative analysis of insulin- (β-cells), glucagon- (α-cells), somatostatin- (δ-cells) and CD31- (endothelial) positive cells (in % of all islet cells) in WT and *AIM2*$^{-/-}$ islets within the pancreas (n = 20 each). Mean ± SEM. (**c**) Representative immunohistochemical stainings of MPO-, CD68- and CD3-positive cells in WT and *AIM2*$^{-/-}$ islets within the pancreas. Scale bar: 50 µm. (**d**) Quantitative analysis of MPO-, CD68- and CD3-positive cells (in % of all islet cells) in WT and *AIM2*$^{-/-}$ islets within the pancreas (n = 20 each). Mean ± SEM.

Figure S2

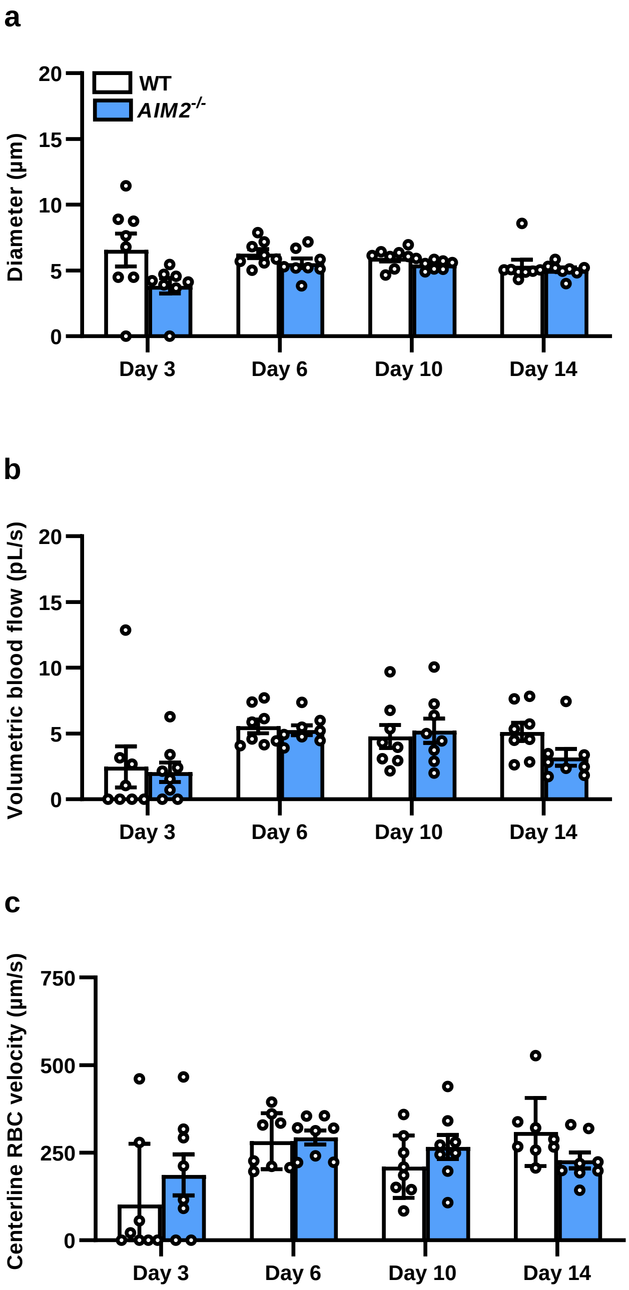

Figure S2. Microhemodynamic parameters of transplanted islets. (**a**-**c**) Quantitative analysis of microvessel diameters (µm) (a), centerline RBC velocities (µm/s) (b) and volumetric blood flows (pL/s) (c) within WT and *AIM2*$^{-/-}$ islets transplanted onto the exposed striated muscle tissue (n = 8 each). Mean ± SEM.

Figure S3

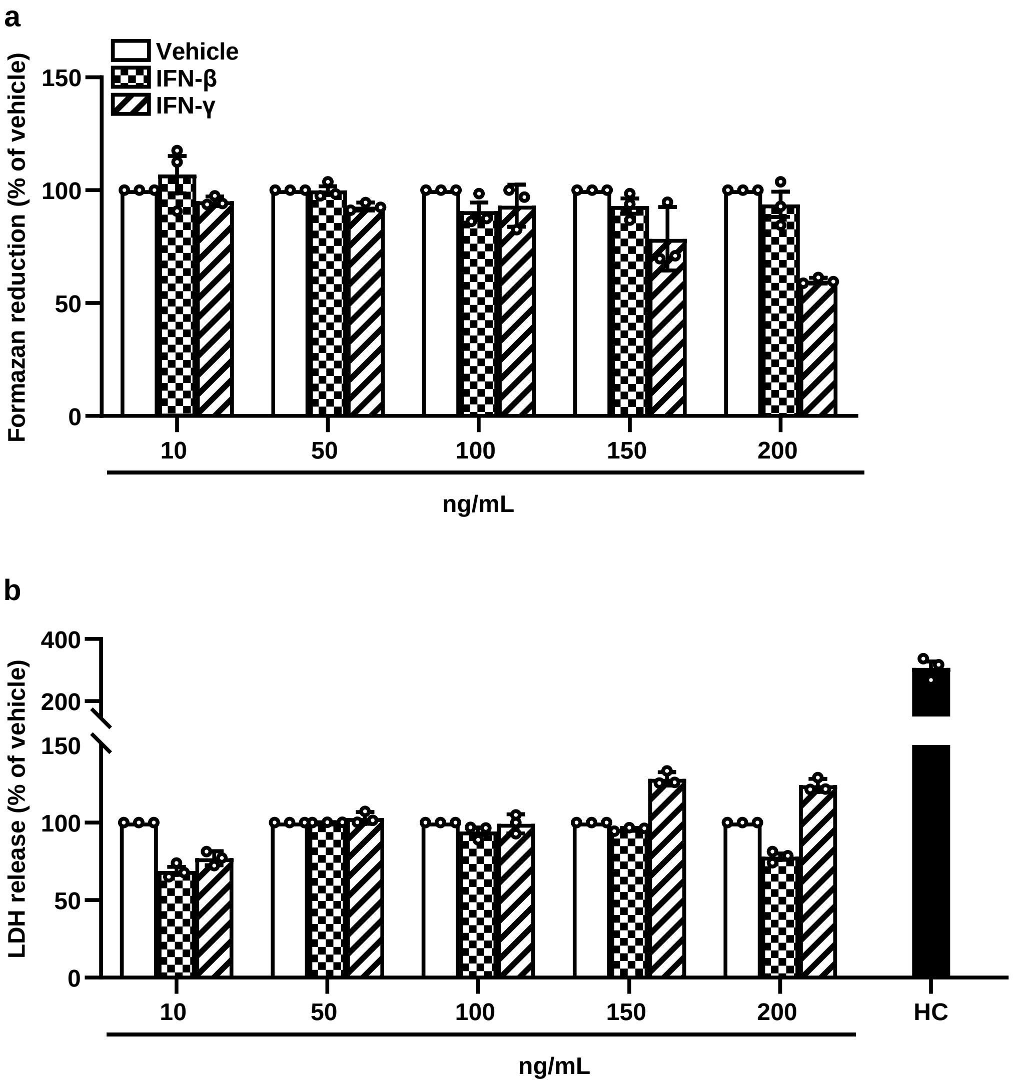

Figure S3. Effect of IFN-β and IFN-γ on the proliferation of HUVEC. (**a**) HUVEC were treated with vehicle, IFN-β or IFN-γ for 24 h and the mitochondrial activity was analyzed by a WST-1 assay. Data are expressed in % of vehicle (n = 3 each). Mean ± SEM. (**b**) HUVEC were treated as described in (a) and the cytotoxicity was assessed by a LDH assay. Data are expressed in % of vehicle (n = 3 each). Cells were permeabilized with Triton-X100 (0.1%) and used as high toxicity control (HC) for the LDH assay (n = 3 each). Mean ± SEM.

Figure S4

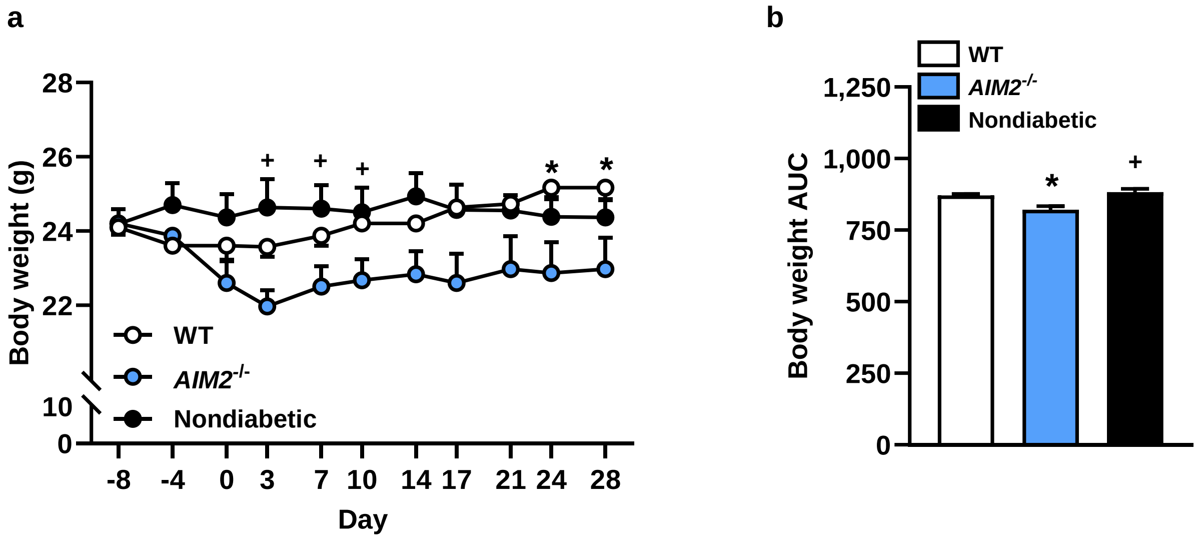

Figure S4. Body weights of islet transplanted mice. (**a**) Quantitative analysis of the body weights (g) of mice transplanted with WT and *AIM2*$^{-/-}$ islets (n = 6 each). Nondiabetic mice served as negative control (n = 6 each). Mean ± SEM. *P < 0.05 vs. WT; $^{+}$P < 0.05 vs. *AIM2*$^{-/-}$. (**b**) AUC of the body weights from (a) (n = 6 each). Mean ± SEM. *P < 0.05 vs. WT; $^{+}$P < 0.05 vs. *AIM2*$^{-/-}$.
